# Predictive Value of Epicardial Adipose Tissue Parameters Measured by Cardiac Computed Tomography for Recurrence of Atrial Fibrillation After Pulmonary Vein Isolation

**DOI:** 10.3390/jcm14196963

**Published:** 2025-10-01

**Authors:** Karol Momot, Michal Pruc, Dariusz Rodkiewicz, Edward Koźluk, Kamil Krauz, Agnieszka Piątkowska, Zuzanna Zalewska, Małgorzata Buksińska-Lisik, Lukasz Szarpak, Artur Mamcarz

**Affiliations:** 1 Chair and Department of Experimental and Clinical Physiology, Medical University of Warsaw, 02-097 Warsaw, Poland; karol.momot@wum.edu.pl (K.M.);; 2 3rd Department of Internal Diseases and Cardiology, Międzylesie Specialist Hospital in Warsaw, Medical University of Warsaw, 04-749 Warsaw, Polandartur.mamcarz@wum.edu.pl (A.M.); 3 Department of Clinical Research and Development, LUXMED Group, 02-678 Warsaw, Poland; 4 Doctoral School, Medical University of Warsaw, 02-097 Warsaw, Poland; 5 Institute of Medical Science, Collegium Medicum, The John Paul II Catholic University of Lublin, 20-950 Lublin, Poland; 6 Henry JN Taub Department of Emergency Medicine, Baylor College of Medicine, Houston, TX 77030, USA

**Keywords:** catheter ablation, atrial fibrillation, epicardial adipose tissue, computed tomography

## Abstract

**Background:** Despite advances in ablation strategies, a substantial proportion of patients with atrial fibrillation (AF) experience arrhythmia recurrence, highlighting the need for improved preprocedural risk stratification. One of the emerging factors associated with arrhythmogenic remodeling is epicardial adipose tissue (EAT), particularly in the proximity of the left atrium (LA), due to its metabolic and inflammatory activity. **Methods:** This study investigated whether preprocedural assessment of EAT parameters on computed tomography (CT), including volume, mean attenuation, and attenuation dispersion, could predict AF recurrence following ablation. Seventy patients with AF underwent either pulsed field or cryoballoon ablation and were followed for 18 months. **Results:** Recurrence of AF occurred in 26 (37.1%) patients. Both higher LA-EAT attenuation (OR 1.09; 95% CI: 1.02–1.17) and greater total-EAT volume (OR 2.41; 95% CI: 1.16–4.99) were independently associated with arrhythmia recurrence. Subgroup analysis revealed that LA-EAT volume was highly predictive of recurrence in patients with persistent AF (AUC = 0.91), whereas LA-EAT attenuation demonstrated greater prognostic value in those with paroxysmal AF (AUC = 0.80). **Conclusions:** These findings suggest that quantitative evaluation of EAT using routine cardiac CT may enhance risk stratification before ablation.

## 1. Introduction

Among sustained cardiac arrhythmias, atrial fibrillation (AF) is the most common in routine clinical practice [1]. In 2020, its global prevalence was estimated to exceed 50 million cases, although the actual burden is likely underestimated due to the presence of asymptomatic and undiagnosed episodes [2]. The condition is associated with significant clinical consequences such as a 3.5-fold increase in mortality risk, decreased tolerance of exercise, left ventricular dysfunction, and heart failure [3]. Despite advances in pulmonary vein isolation (PVI) techniques, its long-term efficacy remains limited: AF recurs in nearly half of patients within five years after a single procedure, and durable sinus rhythm is maintained in only about 30% of cases despite multiple procedures [4]. However, predicting which patients are at risk of AF recurrence after ablation remains a major clinical challenge. Therefore, it is essential to identify factors that impact recurrence to improve patient selection and treatment outcomes. Recent evidence indicates that delayed ablation, persistent AF, advanced age, enlarged left atrium (LA), and comorbidities such as heart failure, hypertension, diabetes, and ischemic heart disease are all associated with increased recurrence risk [5]. One of the factors investigated in our study is epicardial adipose tissue (EAT), which is the fat tissue situated directly between the myocardium and the visceral layer of the pericardium [6]. EAT has been linked to the development and progression of cardiovascular conditions, including AF [7]. It plays an active role in cardiac inflammation by secreting pro-inflammatory mediators, such as adiponectin and adrenomedullin, as well as anti-inflammatory factors, including TNF-α, IL-6, and MCP-1 [8]. The interactions between EAT and myocardium play a pivotal role in generating arrhythmias. This happens through both structural disruption of myocardial conduction pathways and paracrine signalling, which alters ion channel activity, electrical coupling, and induces fibrosis [9].

Numerous studies have examined the association between EAT volume and AF recurrence after ablation, and their findings have been synthesized in three meta-analyses [10,11]. Two of these suggest that a greater total EAT volume or thickness may be linked to higher rates of AF recurrence. However, a more recent analysis found that only EAT volume localized specifically around the LA, rather than total EAT volume, was significantly associated with post-ablation AF recurrence. These findings suggest that the pathophysiological role of EAT may vary depending on its anatomical distribution. Several pathophysiological mechanisms may explain the association between inflamed LA-EAT and AF recurrence following ablation. EAT secretes proinflammatory adipokines that may exert direct paracrine effects on the adjacent atrial myocardium [12]. Increased local inflammation can lead to atrial wall edema, potentially impairing effective lesion formation during ablation [13]. Moreover, EAT-induced inflammation has been implicated in the development of atrial fibrosis [14], which facilitates the formation of re-entry circuits and reduces the efficacy of ablation [13]. Recent studies evaluating various energy settings for AF ablation suggest that optimizing power delivery may enhance lesion durability [15]. This consideration may be particularly relevant in the context of EAT, as increased fat volume and local inflammation can interfere with lesion formation and contribute to arrhythmia recurrence. This may be particularly relevant in patients with greater EAT burden, as increased fat volume and inflammation can interfere with effective lesion formation and promote arrhythmia recurrence.

Importantly, EAT volume is not the only parameter that can be evaluated via preprocedural CT. A relatively novel imaging biomarker, EAT attenuation, has been adapted from approaches used to assess inflammation in perivascular adipose tissue (PVAT) [16,17]. Evidence suggests that EAT attenuation, as measured by cardiac CT, may reflect local inflammatory activity, correlating with histological findings and positron emission tomography (PET) imaging [15]. Inflammatory processes are known to increase EAT attenuation, shifting its Hounsfield Unit (HU) values toward less negative ranges [18]. Given the close anatomical relationship between LA-EAT and the atrial myocardium, the targeted assessment of LA-EAT attenuation may offer a more precise method for evaluating inflammation in this region. Moreover, attenuation measurements appear to be more robust and reproducible than volume-based assessments, which are subject to significant inter-operator variability, particularly in terms of selecting image slices and defining anatomical borders [19,20]. Furthermore, attenuation dispersion, which reflects EAT heterogeneity, may represent an additional promising parameter. However, research in this area remains scarce, and both the definition and methodology for quantifying attenuation heterogeneity have yet to be standardized or validated [21].

While CT-based imaging offers robust quantification of epicardial adipose tissue, cardiac magnetic resonance imaging (MRI) provides complementary insights into atrial structure and function. In particular, MRI enables the assessment of atrial fibrosis and remodeling which are key contributors to AF recurrence. As summarized in the review by Jabbour et al., MRI-derived fibrosis quantification may improve patient stratification and guide substrate-based ablation approaches [22]. However, its broader clinical application remains limited by technical and standardization challenges.

The aim of this study was to determine whether preprocedural measurement of EAT attenuation by cardiac CT can serve as a reliable predictor of ablation outcomes in patients with AF.

## 2. Materials and Methods

### 2.1. Study Population

This single-center, observational, retrospective cohort study included consecutive patients referred for catheter ablation of symptomatic, drug-refractory AF between 1 January 2022, and 31 December 2023, in whom transoesophageal echocardiography (TEE) was not performed due to medical contraindications, insufficient procedural tolerance, or organizational limitations. As an alternative, cardiac computed tomography (CT) was performed to exclude LA appendage (LAA) thrombus and obtain a detailed understanding of LA anatomy. All patients had completed an 18-month clinical follow-up before the analysis of the data. Blood samples were collected as part of standard laboratory workup at the time of hospital admission. Transthoracic echocardiography was performed using the EPIQ Elite CVx system (Philips Healthcare, Andover, MA, USA) at approximately the same time as cardiac CT to assess cardiac chamber dimensions and function. Written informed consent was obtained from all patients before participation, in accordance with routine clinical care procedures. The study protocol was reviewed and approved by the local Ethics Committee of the Medical University of Warsaw (no. AKBE/177/2025; approved on 9 June 2025) and conforms to the ethical guidelines of the Declaration of Helsinki.

### 2.2. CT Acquisition and Image Analysis

All patients underwent cardiac CT using a 64-slice scanner (Philips Incisive CT). Patients with a heart rate greater than 65 beats per minute received beta blockers before image acquisition. A four-phase contrast injection protocol was employed, involving the administration of 85–95 mL of iodinated contrast agent at a rate of 4.5–5.5 mL/s. CT images were acquired with prospective ECG-gating during mid-diastole. The segmentation of EAT was performed semi-automatically, with all contours visually reviewed and manually adjusted if necessary. Total EAT was defined as the adipose tissue enclosed within the visceral pericardium, extending from the level of the pulmonary artery bifurcation to the level of the coronary sinus on axial slices. LA-EAT was defined as the fat surrounding the LA, from the level of the LAA to the superior border of the coronary sinus in the transverse plane. In both defined regions, total-EAT and LA-EAT, the following parameters were measured: volume (in cm^3^), mean attenuation (in HU), and attenuation dispersion, defined as the standard deviation of voxel-wise attenuation values within the segmented region. A predefined attenuation threshold between −200 and −50 HU was applied for all fat segmentation and volumetric analyses.

### 2.3. Procedure Characteristics and Clinical Follow-Up

All procedures were performed using single-shot PVI techniques, either pulsed field ablation (PFA) or cryoballoon ablation (CBA). The choice of ablation modality was determined by the pre-scheduled ablation day, with patients allocated to either PFA or CBA based on the institutional workflow. This allocation was independent of patient characteristics or operator preference. All PFA procedures were performed under general anaesthesia, while all CBA procedures were performed under conscious sedation. In all PFA cases, a Faradrive steerable sheath and a Farawave multispline ablation catheter (Boston Scientific, Marlborough, MA, USA) were used. In CBA cases, a POLARSHEATH steerable sheath, a POLARMAP circular mapping catheter, and a 28-mm POLARx cryoballoon (Boston Scientific, Marlborough, MA, USA) were used. Additionally, diagnostic catheters—a decapolar and a quadripolar electrode catheter (Hagmed, Rawa Mazowiecka, Poland) were used.

Patients underwent follow-up at 3, 6, 12, and 18 months post-ablation, either through in-person outpatient visits or by submitting ECG and Holter results remotely. Follow-up included a 12-lead ECG and 24- or 48-h Holter monitoring, scheduled at 6, 12, and 18 months after procedure. Atrial arrhythmia recurrence (AF or atrial tachycardia) was defined as any documented episode lasting more than 30 s that occurred beyond the 3-month post-ablation blanking period.

### 2.4. Statistical Analysis

All statistical analyses were performed utilizing R version 4.3.1 (R Foundation for Statistical Computing, Vienna, Austria). Continuous variables were expressed as mean with standard deviation (SD) for normally distributed data or as median with interquartile range (IQR) for non-normally distributed data, as determined by the Shapiro–Wilk test. Group comparisons were conducted using the independent-samples t-test for normally distributed variables or the Mann–Whitney U test for non-normally distributed variables. Categorical variables were presented as frequencies and percentages and analyzed using the χ^2^ test or Fisher’s exact test, as applicable. First, univariate logistic regression was used to look at relationships between AF recurrence and clinical, laboratory, imaging, and pharmacological factors. For inclusion in a multivariable model, variables with *p* < 0.10 were taken into consideration. We used LASSO regularization to prevent overfitting and kept a small number of clinically significant predictors. Three variables were included in the final logistic regression model: LA-EAT attenuation (HU), total EAT volume (cm^3^), and LA diameter (mm). The median was used to impute missing data. Odds ratios (ORs) are presented for clinically significant increments (5 HU, 25 cm^3^, 5 mm) and for each 1 SD increase. Multicollinearity (variance inflation factor, VIF), calibration (Hosmer–Lemeshow test), and discrimination (AUC with 5-fold cross-validation) were used to assess the model’s performance. The Benjamini–Hochberg false discovery rate (FDR) method was used to account for multiple testing in univariate analyses, with corrected q < 0.05 being regarded as statistically significant. Spearman’s rank coefficient (ρ) was used to evaluate correlations between continuous predictors and AF recurrence. The correlations were classified as negligible (|ρ| < 0.2), weak (0.2–0.39), moderate (0.4–0.59), or strong (≥0.6). All statistical tests were bilateral, with significance established at *p* < 0.05 unless stated otherwise.

## 3. Results

### 3.1. Patient Population

Baseline characteristics of the study population are presented in Table 1. A total of 70 patients were included, with a mean age of 62.97 (9.54) years. Women accounted for 28.6% of the cohort. The mean left ventricular ejection fraction (LVEF) was 57.41 (8.43) %, and the mean body mass index (BMI) was 29.07 (4.13) kg/m^2^. Fifty-six patients (80%) were diagnosed with paroxysmal AF. Findings from cardiac CT, including quantitative assessment of EAT volume and attenuation, are summarized in Table 2.

### 3.2. Procedural Characteristics and Ablation Outcomes

Details regarding the ablation procedure are presented in Supplementary Table S1. A low rate of procedure-related complications was observed. Reported adverse events included one case of pericardial effusion, one groin complication in the form of an arteriovenous fistula, and one case of phrenic nerve palsy. Notably, no cases of atrio-esophageal fistula, cardiac arrest, or periprocedural death occurred in the study cohort. At the 18-month follow-up, freedom from atrial fibrillation or atrial tachycardia was observed in 44 out of 70 patients (62.9%), excluding episodes within the 30-day post-ablation blanking period and considering only documented episodes lasting more than 30 s.

### 3.3. EAT in “Recurrence” and “Non-Recurrence” Groups

Patients with AF recurrence had significantly greater LA diameter (44.67 (4.21) vs. 40.50 (4.50) mm; *p* < 0.001), as well as increased EAT volumes and attenuation values, both in total and in the proximity of the LA. Specifically, LA-EAT volume was markedly higher in the recurrence group (26.83 (14.26) vs. 18.90 (8.79) cm^3^; *p* = 0.022), and LA-EAT attenuation values were significantly less negative (−89.69 (4.14) vs. −93.91 (3.95) HU; *p* < 0.001). Similarly, total EAT volume was significantly increased in patients with AF recurrence (120.0 (56.2) vs. 75.75 (27.03) cm^3^; *p* = 0.041) (Table 1).

### 3.4. Univariate Logistic Regression

In univariate logistic regression analysis, multiple parameters were found to be substantially correlated with the recurrence of AF. The factors were LA-EAT attenuation (OR 1.29; 95% CI 1.12–1.50; *p* < 0.001), total-EAT volume (OR 1.03; 95% CI 1.01–1.04; *p* < 0.001), LA diameter (OR 1.26; 95% CI 1.09–1.46; *p* = 0.002), BMI (OR 1.20; 95% CI 1.05–1.38; *p* = 0.0088), and body weight (OR 1.05; 95% CI 1.01–1.09; *p* = 0.0137). Increased dispersion of EAT attenuation was associated with a reduced risk of recurrence for both LA-EAT (OR 0.79; 95% CI 0.68–0.92; *p* = 0.002) and total-EAT (OR 0.82; 95% CI 0.71–0.95; *p* = 0.010) (Supplementary Table S2).

The Spearman correlation coefficients validated the aforementioned conclusions. The most robust positive relationships with recurrence were noted for total-EAT volume (ρ = 0.47), LA-EAT attenuation (ρ = 0.46), and LA diameter (ρ = 0.43). Conversely, AF type (paroxysmal vs. persistent) exhibited a weak inverse correlation (ρ = −0.28), indicating reduced recurrence rates in patients with paroxysmal AF (Supplementary Figure S1).

### 3.5. Multivariate Logistic Regression Analysis

The final multivariable logistic regression model (N = 70, events = 26) included three predictors: LA-EAT attenuation, whole-EAT volume, and LA diameter. Both LA-EAT attenuation (OR per 1 SD 3.34, 95% CI 1.46–7.64, *p* = 0.004) and LA diameter (OR per 1 SD 2.91, 95% CI 1.29–6.58, *p* = 0.010) were independently associated with arrhythmia recurrence, while whole-EAT volume showed a borderline association (OR per 1 SD 1.93, 95% CI 0.93–4.02, *p* = 0.078). The model achieved an AUC of 0.878 in-sample and 0.876 ± 0.087 with 5-fold cross-validation, with acceptable calibration (Hosmer–Lemeshow *p* = 0.085). All predictors had VIF < 1.4, confirming the absence of relevant collinearity.

Table 3 presents the final multivariable logistic regression model including LA-EAT attenuation, whole-EAT volume, and LA diameter. Supplementary Table S3 reports the full regression coefficients for the final 3-predictor logistic regression model, including log-odds coefficients, standard errors (SE), *p*-values, and odds ratios (ORs) per 1 SD and per clinically meaningful increments.

### 3.6. Analysis of ROC Curves for LA-EAT Parameters

**Figure 1:**
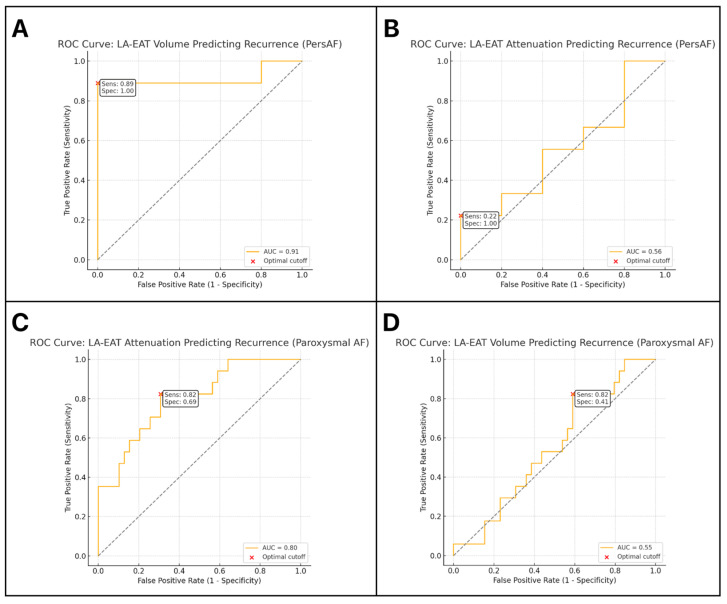
Receiver operating characteristic (ROC) curves for LA-EAT volume and attenuation in predicting atrial arrhythmia recurrence after pulmonary vein isolation (PVI) in patients with persistent (PersAF) and paroxysmal atrial fibrillation (AF). (**A**) LA-EAT volume—PersAF (**B**) LA-EAT attenuation—PersAF (**C**) LA-EAT attenuation—Paroxysmal AF (**D**) LA-EAT volume—Paroxysmal AF. Abbreviations: AF, Atrial Fibrillation; LA-EAT, Left Atrium Epicardial Adipose Tissue; PVI, Pulmonary Vein Isolation; ROC, Receiver Operating Characteristic; PersAF, Persistent Atrial Fibrillation.

The LA-EAT volume demonstrates very high diagnostic value in predicting AF recurrence after ablation in patients with persistent AF. AUC = 0.91 suggests excellent discriminative power. A sensitivity of 89% indicates that this parameter efficiently identifies patients with a high risk of AF recurrence, and a specificity of 100% indicates that there are no false-positive cases (Figure 1A).

Despite the high sensitivity (82%), low AUC (0.55), and very low specificity (41%), these results suggest that the LA-EAT volume has limited predictive value in patients with paroxysmal AF. The high percentage of false-positive cases limits the clinical utility of this test in this patient subgroup (Figure 1B).

Despite ideal specificity, LA-EAT attenuation exhibits very low sensitivity (22%) and an AUC close to random (0.56). This means that LA-EAT attenuation is not clinically useful in predicting AF recurrence following ablation in patients with persistent AF (Figure 1C).

In the subset of patients with paroxysmal AF, LA-EAT attenuation was an efficient predictor of AF recurrence (AUC = 0.80). High sensitivity (82%) suggests its usefulness in identifying individuals at risk of AF recurrence. Even though the specificity is moderate, this parameter may hold potential clinical significance in this subgroup of patients (Figure 1D).

## 4. Discussion

This study provides new evidence supporting the predictive value of preprocedural CT-based EAT parameters in patients undergoing PVI for AF. Among the evaluated metrics, higher (less negative) LA-EAT attenuation and increased total EAT volume were independently associated with arrhythmia recurrence, suggesting that both inflammatory activity and structural fat burden may contribute to ablation failure. Although LA-EAT volume was significantly associated with AF recurrence in univariate analysis, this association was not retained in the multivariable model—likely due to collinearity with other structural markers, particularly LA diameter and total EAT volume, which capture overlapping aspects of atrial remodeling. While LA-EAT volume may still hold clinical relevance, its independent predictive value appears diminished when these related variables are considered concurrently.

ROC curve analysis revealed a differential diagnostic performance of EAT parameters across AF subtypes. In persistent AF, LA-EAT volume demonstrated excellent discriminative power, with an AUC of 0.91, sensitivity of 89%, and perfect specificity (100%), highlighting its potential role as a robust predictor of recurrence in this subgroup. In contrast, its performance in paroxysmal AF was limited (AUC 0.55), primarily due to its low specificity (41%), despite a high sensitivity (82%), which may restrict its utility in this population. Conversely, LA-EAT attenuation was more useful in paroxysmal AF, with an AUC of 0.80 and a sensitivity of 82%, suggesting its relevance in identifying patients at higher risk of recurrence, which may reflect underlying local inflammation; however, this association requires further prospective validation. In persistent AF, however, attenuation was not clinically informative (AUC 0.56, sensitivity 22%), possibly because, with disease progression, arrhythmia maintenance becomes increasingly driven by structural remodeling rather than EAT-related metabolic activity. This hypothesis warrants further investigation.

Our findings are consistent with our recently published meta-analysis, in which we demonstrated that LA-EAT attenuation is significantly more positive in patients experiencing AF recurrence compared to those without recurrence following ablation procedures [23]. However, that analysis was limited to comparing mean attenuation values due to the insufficient number of studies reporting odds ratios. This limitation provided the rationale for the present study, which aims to address this gap by offering multivariate risk estimates. Among the limited number of available reports, our results align with those published by Yang et al., who demonstrated that LA-EAT attenuation remained independently associated with AF recurrence in multivariate logistic regression analysis [24].

It is essential to consider the method of LA-EAT assessment, as evidence suggests that a comprehensive 3D approach surrounding the entire LA may be more optimal. Ciuffo et al. employed a 2D approach, manually segmenting LA-EAT in standard 4-chamber and 2-chamber views. They found that mean attenuation in the 4-chamber view differed significantly between patients with and without recurrence, whereas no difference was observed in the 2-chamber view [25]. In contrast, Mahdiui et al., despite using a 3D method, focused exclusively on posterior LA-EAT and did not observe a significant difference between groups [18]. These findings are also in line with our previous meta-analysis [23], emphasizing the importance of comprehensive, 3D quantification of LA-EAT attenuation. While simplified 2D or region-specific approaches may offer practical advantages in busy clinical settings, they risk introducing variability and limiting diagnostic accuracy.

Another important limitation is the lack of consensus regarding the definition and segmentation boundaries of LA-EAT, which hinders direct comparisons between studies. In our analysis, we applied the most commonly used anatomical boundaries, extending from the left atrial appendage to the coronary sinus. Nevertheless, broader methodological standardization is urgently needed. Although attenuation measurements are generally more reproducible than volumetric assessments, inter-operator variability may still influence results. We applied the −50 to −200 Hounsfield Unit (HU) threshold in accordance with most prior studies on adipose tissue imaging. However, alternative thresholds, such as −190 HU or −30 HU, have also been reported. This variability underscores the need for unified imaging protocols and consistent attenuation thresholds across studies. The harmonization of CT acquisition protocols and attenuation thresholds across centers, supported by expert consensus and multicenter collaboration, may help to clarify the role of LA-EAT attenuation in future personalized risk stratification strategies for AF patients. Importantly, it must be acknowledged that LA-EAT attenuation remains a surrogate marker of local inflammation, and in the absence of histological or PET-based validation, the biological interpretation of these findings remains speculative.

Our results raise the question of whether, in patients with confirmed high LA-EAT inflammatory activity, PVI alone is sufficient or whether additional interventions targeting LA-EAT should be considered. There is evidence that specific pharmacological agents may modulate both EAT inflammation and volume. These include statins, SGLT2 inhibitors, and incretin-based therapies such as GLP-1 receptor agonists [26,27,28]. Beyond pharmacotherapy, lifestyle interventions are equally important. Regular physical activity and intentional weight loss have been shown to reduce both the volume and inflammatory activity of visceral fat depots, including those associated with EAT [29,30]. Interestingly, many of these interventions have also been independently associated with improved PVI outcomes and a reduced risk of AF recurrence. Although no study has directly evaluated whether these benefits are mediated through modulation of EAT, the overlap in therapeutic effects suggests that EAT may represent a mechanistic link between metabolic interventions and arrhythmia burden. A meta-regression analysis demonstrated that each 1% absolute reduction in body weight was associated with a 6% relative risk reduction in AF recurrence (RR = 0.94; 95% CI: 0.90–0.98), irrespective of the weight loss strategy applied [31]. Regarding pharmacotherapy, a recent meta-analysis found that the use of GLP-1 receptor agonists was associated with a reduced risk of AF recurrence in patients undergoing ablation therapy [32]. Moreover, in obese individuals, GLP-1 receptor agonists have been linked to a significantly lower composite risk of cardioversion, new antiarrhythmic drugs initiation, repeat ablation, as well as reduced AF- and HF-related readmissions and all-cause mortality. However, not all findings are consistent: a separate analysis reported that 1-year preprocedural use of GLP-1 receptor agonists did not result in reduced risk of AF recurrence or related complications following ablation [33], underlining the need for further prospective trials. Although no published data are yet available on the effectiveness of tirzepatide (a dual GLP-1/GIP receptor agonist) in reducing post-ablation AF recurrence, a recent study showed that its use in patients with T2D and AF was associated with a significant reduction in AF burden (HR 0.65; 95% CI: 0.55–0.76). The composite primary endpoint in this study included cardioversion, the use of intravenous antiarrhythmic drugs, and AF ablation [34]. SGLT2 inhibitors also demonstrate consistent benefits. A meta-analysis of seven randomized controlled trials (RCTs) reported that SGLT2i use after AF ablation was associated with a 39% reduction in AF recurrence (RR 0.61; 95% CI: 0.49–0.77) [34]. Additional analyses confirmed a significant reduction in AF recurrence among diabetic patients receiving SGLT2i compared to those on other glucose-lowering therapies (RR 0.72; 95% CI: 0.67–0.78) and documented fewer hospitalizations and ischemic strokes [35]. Moreover, direct meta-analyses showed that SGLT2i use was associated with a lower risk of AF recurrence compared to both all antidiabetic drugs (HR 0.57; 95% CI: 0.44–0.73) and dipeptidyl peptidase-4 (DPP4) inhibitors (HR 0.41, 95% CI: 0.24–0.70) [36]. Following ablation, SGLT2 inhibitor medication is linked to a considerable decrease in AF recurrence, according to a more recent arrhythmia-focused meta-analysis [37]. Finally, evidence regarding statin therapy is mixed. While a meta-analysis found that statin use significantly reduced AF recurrence in RCTs (OR 0.47; 95% CI: 0.30–0.75), this effect was not confirmed when including observational studies (OR 0.81; 95% CI: 0.59–1.10) [38]. Furthermore, different ablation modalities may provoke distinct inflammatory responses, potentially influencing arrhythmia recurrence irrespective of the initial EAT profile, underscoring the need for future studies exploring these relationships. These findings highlight the therapeutic potential of metabolic modulation (both pharmacological and lifestyle-based) in patients undergoing AF ablation. While growing evidence supports the use of agents targeting inflammation and metabolic dysfunction, prospective studies directly linking EAT modulation with improved ablation outcomes remain critically needed.

### Limitations

This study has several limitations that should be acknowledged. Its retrospective, single-center design and comparatively small sample size may have limited its statistical power and generalizability. The stability of regression models may also be impacted by the small number of outcome events relative to predictors. Our findings should be considered hypothesis-generating and need to be confirmed in larger, multicenter prospective cohorts, even though we used cross-validation and regularization to reduce overfitting. Secondly, analyses of subgroups by type of AF lacked sufficient power. It’s possible that sample-size artifacts rather than actual discriminative capacity are responsible for the extremely high AUC values seen in some subgroups, especially persistent AF. Therefore, these findings should be regarded as exploratory and should be confirmed in populations with adequate power. Third, formal testing of interaction effects, like those between EAT parameters and AF subtype, was not possible due to the small sample size. Although stratified ROC analyses were conducted, larger cohorts would be necessary for robust interaction analyses. Lastly, another methodological constraint is the lack of agreed-upon definitions for segmentation boundaries and attenuation thresholds for LA-EAT.

## 5. Conclusions

Preprocedural measurement of EAT parameters by cardiac CT can serve as a reliable predictor of PVI outcomes in patients with AF. In particular, higher (less negative) LA-EAT attenuation and increased total-EAT volume were independently associated with arrhythmia recurrence. These findings suggest that quantitative EAT assessment holds potential for future clinical application; however, prospective studies are warranted to validate its predictive utility in routine practice and to investigate whether interventions targeting EAT can improve PVI outcomes.

## Figures and Tables

**Table 1:** Baseline Characteristics of the Study Population.

Variable	Total Population (n = 70)	Non-Recurrence Group (n = 44)	Recurrence Group (n = 26)
Age, years, mean (SD)	62.97 (9.54)	61.82 (10.25)	64.92 (8.00)
Female sex, n (%)	20 (28.6%)	14 (31.8%)	6 (23%)
BMI, kg/m^2^, mean (SD)	29.07 (4.13)	28.02 (3.50)	30.83 (4.57)
Echocardiography features			
LVEF, %, mean (SD)	57.41 (8.43)	58.85 (7.36)	55.08 (9.66)
LA diameter, millimeters, mean (SD)	42.11 (4.80)	40.50 (4.50)	44.66 (4.20)
Comorbidities			
Persistent AF, n (%)	14 (20%)	5 (11.4%)	9 (34.6%)
Diabetes mellitus, n (%)	14 (20%)	8 (18.2%)	6 (23.1%)
HFpEF, n (%)	7 (10%)	2 (4.5%)	5 (19.2%)
HFrEF, n (%)	5 (7.1%)	3 (6.8%)	2 (7.7%)
CAD, n (%)	10 (14.3%)	5 (11.4%)	5 (19.2%)
Hypertension, n (%)	44 (62.9%)	24 (54.5%)	20 (77%)
Thyroid disease, n (%)	21 (30%)	12 (27.3%)	9 (33.3%)
Dyslipidemia, n (%)	42 (60%)	25 (56.8%)	17 (65.4%0
Obesity, n (%)	26 (37.1%)	12 (27.3%)	14 (53.8%)
OSA, n (%)	2 (2.8%)	2 (4.5%)	0 (0%)
COPD, n (%)	1 (1.4%)	0 (0%)	1 (3.8%)
Asthma, n (%)	3 (4.3%)	1 (2.3%)	2 (7.7%)
Smoking	10 (14.3%)	4 (9.1%)	6 (23.1%)
Previous PCI/CABG, n (%)	4 (5.7%)	4 (9.1%)	0 (0%)
Preprocedural laboratory variables			
Creatinine, mg/L, mean (SD)	82.80 (17.05)	81.39 (15.97)	85.19 (18.81)
CRP, mg/L, median (IQR)	3.92 (9.93)	2.36 (2.79)	7.12 (16.68)
TSH, uIU/mL, median (IQR)	1.45 (1.13, 2.23)	1.69 (1.25, 2.37)	1.43 (1.06, 2.2)
eGFR, mL/min/1.73, mean (SD)	81.18 (15.50)	82.76 (14.33)	78.52 (17.28)
Medications			
GLP1ra, n (%)	3 (4.3%)	0 (0%)	3 (11.5%)
SGLT2i, n (%)	11 (15.7%)	5 (11.4%)	6 (23.1%)
BB, n (%)	51(72.8%)	30 (68.2%)	21(80.8%)
CaB, n (%)	13 (18.6%)	6 (13.6%)	7 (26.9%)
ACEI/ARB, n (%)	45 (64.3%)	25 (56.9%)	20 (77%)
Loop diuretics, n (%)	9 (12.9%)	5 (11.4%)	4 (15.4%)
Thiazide diuretics, n (%)	12 (17.1%)	4 (9.1%)	8 (30.8%)
MRA, n (%)	16 (22.9%)	11 (25%)	5 (19.2%)
VKA, n (%)	3 (4.3%)	1 (2.3%)	2 (7.7%)
NOAC, n (%)	63 (90%)	40 (91%)	23 (88.5%)
Levothyroxine, n (%)	14 (20%)	8 (18.1%)	6 (22.2%)
Metformin, n (%)	9 (12.9%)	5 (11.4%)	4 (15.4%)
Insulin, n (%)	2 (2.8%)	1 (2.3%)	1 (3.8%)
Antiarrhythmic drugs, n (%)	15 (21.4%)	7 (16%)	8 (30.8%)
Statines, n (%)	53 (75.7%)	31 (70.5%)	22 (84.6%)

Abbreviations: ACEI, Angiotensin-Converting Enzyme Inhibitor; AF, Atrial Fibrillation; ARB, Angiotensin II Receptor Blocker; BB, Beta Blockers; BMI, Body Mass Index; CaB, Calcium Blockers; CABG, Coronary Artery Bypass Grafting; CAD, Coronary Artery Disease; COPD, Chronic Obstructive Pulmonary Disease; CRP, C-Reactive Protein; GFR, Glomerular Filtration Rate; GLP1ra, Glucagon-Like Peptide-1 Receptor Agonist; HFpEF, Heart Failure with Preserved Ejection Fraction; HFrEF, Heart Failure with Reduced Ejection Fraction; IQR, Interquartile Range; LA, Left Atrium; LVEF, Left Ventricular Ejection Fraction; MRA, Mineralocorticosteroid Receptor Antagonist; NOAC, Non-Vitamin K Antagonist Oral Anticoagulant; OSA, Obstructive Sleep Apnea; PCI, Percutaneous Coronary Intervention; SD, Standard Deviation; SGLT2i, Sodium-Glucose Cotransporter-2 Inhibitor; TSH, Thyroid-Stimulating Hormone; VKA, Vitamin K Antagonist.

**Table 2:** Cardiac CT characteristics of the study population.

Variable	Total Population (n = 70)	Non-Recurrence Group (n = 44)	Recurrence Group (n = 26)
Patients with three pulmonary veins, n (%)	9 (12.8%)	5 (11.4%)	4 (15.4%)
Patients with four pulmonary veins, n (%)	45 (64.3%)	31 (70.4%)	14 (53.8%)
Patients with five pulmonary veins, n (%)	12 (17.1%)	6 (13.6%)	6 (23%)
LA-EAT attenuation, HU, mean (SD)	−92.34 (4.49)	−93.91 (3.95)	−89.69 (4.14)
LA-EAT attenuation dispersion, mean (SD)	34.37 (4.01)	35.58 (3.61)	32.33 (3.89)
LA-EAT volume, cm^3^, median (IQR)	20.14 (12.59, 28.20)	17.96 (11.98, 24.97)	23.67 (14.53, 34.69)
Total-EAT attenuation, HU, median (IQR)	−89.98 (−91.83, −88.21)	−90.10 (−92.56, −88.70)	−89.29 (−91.17, −86.83)
Total-EAT attenuation dispersion, mean (SD)	31.40 (3.87)	32.36 (3.57)	29.79 (3.87)
Total-EAT volume, cm^3^, median (IQR)	80.87 (56.31, 104.44)	72.95 (51.24, 93.23)	99.98 (78.7, 171.74)

Abbreviations: cm^3^, cubic centimetres; HU, Hounsfield Units; IQR, Interquartile Range; LA-EAT, Left Atrium Epicardial Adipose Tissue; SD, Standard Deviation; Total-EAT, Total Epicardial Adipose Tissue.

**Table 3:** Multivariate Logistic Regression for AF Recurrence.

Variable	Odds Ratio (95% CI)	*p*-Value	OR per Clinical Step (95% CI)
LA-EAT attenuation, HU	1.09 (1.02–1.17)	0.01519	3.87 per + 5 HU (1.53–9.81)
Whole-EAT volume, cm^3^	2.41 (1.16–4.99)	0.01773	1.45 per + 25 cm^3^ (0.96–2.19)
LA diameter, mm	1.44 (1.04–2.00)	0.02954	3.28 per + 5 mm (1.32–8.12)

Abbreviations: CI = confidence interval; OR = odds ratio; HU = Hounsfield units; cm^3^ = cubic centimetres; mm = millimetres.

## Data Availability

The data that support the findings of this study are available from the corresponding author upon reasonable request.

## Supplementary Materials

The following supporting information can be downloaded at: https://www.mdpi.com/article/10.3390/jcm14196963/s1. Table S1: Procedural characteristics. Table S2: Univariate logistic regression for AF recurrence. Table S3: Full Logistic Regression Output. Figure S1: Sorted correlation heatmap.

## Abbreviations

The following abbreviations are used in this manuscript:

ACEI	Angiotensin-Converting Enzyme Inhibitor
AF	Atrial Fibrillation
AUC	Area Under the Curve
ARB	Angiotensin II Receptor Blocker
BB	Beta Blockers
BMI	Body Mass Index
CABG	Coronary Artery Bypass Grafting
CaB	Calcium Blockers
Ca-blocker	Calcium Channel Blocker
CAD	Coronary Artery Disease
CCS	Chronic Coronary Syndrome
cm^3^	Cubic Centimetres
CBA	Cryoballoon Ablation
CI	Confidence Interval
COPD	Chronic Obstructive Pulmonary Disease
CRP	C-Reactive Protein
CT	Computed Tomography
DPP4	Dipeptidyl Peptidase-4
EAT	Epicardial Adipose Tissue
ECG	Electrocardiogram
FDR	False Discovery Rate
GFR	Glomerular Filtration Rate
GIP	Glucose-Dependent Insulinotropic Polypeptide
GLP-1	Glucagon-Like Peptide-1
GLP1ra	Glucagon-Like Peptide-1 Receptor Agonist
HF	Heart Failure
HFpEF	Heart Failure with Preserved Ejection Fraction
HFrEF	Heart Failure with Reduced Ejection Fraction
HU	Hounsfield Units
IQR,	Interquartile Range
LA	Left Atrium
LA-EAT	Left Atrium Epicardial Adipose Tissue
LAA	Left Atrial Appendage
LVEF	Left Ventricular Ejection Fraction
MI	Myocardial Infarction
MRA	Mineralocorticoid Receptor Antagonist
NOAC	Non-Vitamin K Antagonist Oral Anticoagulant
OBS	Obstructive Sleep Apnea
OR	Odds Ratio
OSA	Obstructive Sleep Apnea
PCI	Percutaneous Coronary Intervention
PET	Positron Emission Tomography
PersAF	Persistent Atrial Fibrillation
PFA	Pulsed Field Ablation
PVAT	Perivascular Adipose Tissue
PVI	Pulmonary Vein Isolation
RCT	Randomized Controlled Trial
ROC	Receiver Operating Characteristic
SD	Standard Deviation
SGLT2i	Sodium-Glucose Cotransporter-2 Inhibitor
T2D	Type 2 Diabetes
Total-EAT	Total Epicardial Adipose Tissue
TSH	Thyroid-Stimulating Hormone
VKA	Vitamin K Antagonist
