# Supplementary material for: Predictive Value of Epicardial Adipose Tissue Parameters Measured by Cardiac Computed Tomography for Recurrence of Atrial Fibrillation After Pulmonary Vein Isolation
Source: J Clin Med. 2025 Oct 1;14(19):6963. doi: 10.3390/jcm14196963 (PMC12524481; doi:10.3390/jcm14196963)
Supplement: Supplementary file 1 [file jcm-14-06963-s001.zip › Supplementary Tables.pdf]

Supplementary Table 1. Procedural characteristics.

Variable	Total population (n = 70)	Non-recurrence group (n = 44)	Recurrence group (n = 26)
CBA, n (%)	42 (60%)	28 (63.6%)	14 (53.8%)
PFA, n (%)	28 (40%)	16 (36.4%)	12 (46.1%)
Baseline sinus rhythm, n (%)	53 (75.7%)	37 (84.1%)	16 (61.5%)
Procedure time, minutes, median (IQR)	85.0 (66.5, 108.5)	81.5 (65.75, 105.75)	87 (75, 125)
Fluoroscopy time, minutes, median (IQR)	13.61 (10, 18.96)	13.43 (9.85, 17.48)	14.50 (10.79, 20.90)
Cardioversion during procedure, n (%)	14 (20%)	6 (13.6%)	8 (30.8%)
Wenckebach period, milliseconds, mean (SD)	410.95 (76.90)	404.40 (81.40)	438.80 (53.40)
Number of CBA applications, n, mean (SD)	7.17 (2.21)	6.85 (2.36)	7.79 (1.81)
Total CBA application time, minutes, mean (SD)	24.99 (7.43)	23.69 (7.75)	27.50 (6.29)
Number of PFA applications, n, mean (SD)	48.90 (15.00)	44.55 (13.31)	55.71 (15.82)

Abbreviations: CBA, Cryoballoon Ablation; DAP, Dose Area Product; IQR, Interquartile Range; PFA, Pulsed Field Ablation; SD, Standard Deviation.

Supplementary Table 2. Univariate Logistic Regression for AF Recurrence

Variable	Odds Ratio (95% CI)	p-value
LA-EAT attenuation	1.29 (1.12-1.50)	0.00048
Total-EAT volume	1.03 (1.01-1.04)	0.00074
LA diameter	1.26 (1.09-1.46)	0.00202
LA-EAT attenuation dispersion	0.79 (0.68-0.92)	0.00218
BMI	1.20 (1.05-1.38)	0.00881
Total-EAT attenuation dispersion	0.82 (0.71-0.95)	0.00996
LA-EAT volume	1.07 (1.01-1.12)	0.01051
Paroxysmal AF	0.24 (0.07-0.83)	0.02415
Thiazide diuretic treatment	4.44 (1.18-16.69)	0.0271
Obesity	3.11 (1.13-8.60)	0.02875
Total-EAT attenuation	1.18 (1.00-1.39)	0.047
Hypertension	2.78 (0.94-8.25)	0.06578
Fluoroscopy time	1.07 (0.99-1.15)	0.09264
ACEI/ARB treatment	2.53 (0.85-7.54)	0.09464
HF	2.87 (0.81-10.25)	0.10382
LVEF	0.95 (0.88-1.01)	0.10762
Smoking	3.00 (0.76-11.86)	0.11717
Antiarrhythmic drugs treatment	2.35 (0.74-7.50)	0.14909
Preprocedural CRP level	1.13 (0.95-1.34)	0.15889
Ca-bloker treatment	2.33 (0.69-7.92)	0.17401
Preprocedural TSH level	0.71 (0.43-1.17)	0.18255
Statin treatment	2.31 (0.66-8.02)	0.18892
Age	1.04 (0.98-1.10)	0.19101
SGLT2i treatment	2.34 (0.64-8.62)	0.20114
Beta-blocker treatment	1.96 (0.61-6.27)	0.25695
Preprocedural GFR	0.98 (0.95-1.01)	0.27
Asthma	3.58 (0.31-41.60)	0.30761
Preprocedural creatinine level	1.01 (0.98-1.04)	0.36789
CAD	1.86 (0.48-7.15)	0.3682
Ablation method	1.50 (0.56-4.02)	0.42016
Dyslipidemia	1.44 (0.53-3.92)	0.48044
Previous MI	1.78 (0.33-9.56)	0.5
Thyroid diseases	1.41 (0.50-4.02)	0.51796
MRA treatment	0.71 (0.22-2.35)	0.57956
Diabetes Melitus	1.35 (0.41-4.44)	0.62149
Levothyroxine treatment	1.35 (0.41-4.44)	0.62149
Metformin treatment	1.42 (0.34-5.84)	0.62839
Loop diuretic treatment	1.42 (0.34-5.84)	0.62839
Procedure time	1.00 (0.99-1.01)	0.64108
Male sex	1.27 (0.43-3.71)	0.66617
Insulin treatment	1.72 (0.10-28.72)	0.70576

Abbreviations: ACEI, Angiotensin-Converting Enzyme Inhibitor; AF, Atrial Fibrillation; ARB, Angiotensin II Receptor Blocker; BMI, Body Mass Index; CABG, Coronary Artery Bypass Grafting; CAD, Coronary Artery Disease; COPD, Chronic Obstructive Pulmonary Disease; CRP, C-Reactive Protein; GLP1ra, Glucagon-Like Peptide-1 Receptor Agonist; GFR, Glomerular Filtration Rate; HF, Heart Failure; LA, Left Atrium; LA-EAT, Left Atrium Epicardial Adipose Tissue; LVEF, Left Ventricular Ejection Fraction; MI, Myocardial Infarction; MRA, Mineralocorticoid Receptor Antagonists; NOAC, Non-Vitamin K Antagonist Oral Anticoagulant; PCI, Percutaneous Coronary Intervention; SGLT2i, Sodium-Glucose Cotransporter-2 Inhibitor; Total-EAT, Total Epicardial Adipose Tissue; TSH, Thyroid-Stimulating Hormone; VKA, Vitamin K Antagonist.

Supplementary Table S3. Full Logistic Regression Output

Predictor	Coefficient (log-odds)	SE	p-value	OR per 1 SD	95% CI (SD)	OR per clinical step	95% CI (step)	SD	Clinical step
LA-EAT attenuation	0.27	0.09	0.0043	3.34	1.46–7.64	3.87	1.53–9.81	4.45	5.0 HU
Whole-EAT volume	0.01	0.01	0.0784	1.93	0.93–4.02	1.45	0.96–2.19	44.36	25.0 cm^3
LA diameter	0.24	0.09	0.0102	2.91	1.29–6.58	3.28	1.32–8.12	4.50	5.0 mm

Abbreviations: CI, Confidence Interval; cm^3, cubic centimetres; HU, Hounsfield Units; IQR, Interquartile Range; LA-EAT, Left Atrium Epicardial Adipose Tissue; OR, Odds Ratio; SD, Standard Deviation; SE, Standard Error; Total-EAT, Total Epicardial Adipose Tissue.
