# Supplementary figures and images for: Predictive Value of Epicardial Adipose Tissue Parameters Measured by Cardiac Computed Tomography for Recurrence of Atrial Fibrillation After Pulmonary Vein Isolation
Source: J Clin Med. 2025 Oct 1;14(19):6963. doi: 10.3390/jcm14196963 (PMC12524481; doi:10.3390/jcm14196963)

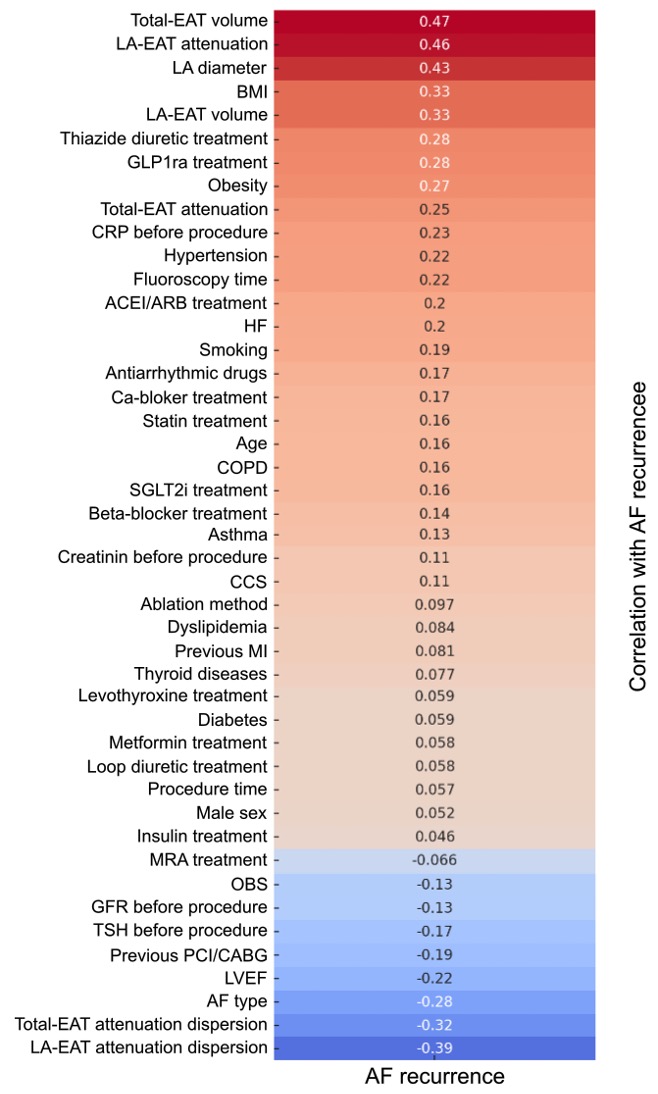

Supplement: Supplementary file 1 [file jcm-14-06963-s001.zip › Supplementary Figure 1.jpeg]
